# Study on Citrus Response to Huanglongbing Highlights a Down-Regulation of Defense-Related Proteins in Lemon Plants Upon ‘*Ca*. Liberibacter asiaticus’ Infection

**DOI:** 10.1371/journal.pone.0067442

**Published:** 2013-06-19

**Authors:** Chika C. Nwugo, Yongping Duan, Hong Lin

**Affiliations:** 1 San Joaquín Valley Agricultural Sciences Center, Department of Agriculture, ARS, Parlier, California, United States of America; 2 U.S. Horticultural Research Laboratory, USDA, ARS, Fort Pierce, Florida, United States of America; University of Wisconsin-Milwaukee, United States of America

## Abstract

Citrus huanglongbing (HLB) is a highly destructive disease of citrus presumably caused by ‘ *Candidatus* Liberibacter asiaticus ’ (Las), a gram-negative, insect-transmitted, phloem-limited α-proteobacterium. Although almost all citrus plants are susceptible to HLB, reports have shown reduced susceptibility to Las infection in lemon ( *Citrus* *limon* ) plants. The aim of this study is to identify intra-species specific molecular mechanisms associated with Las-induced responses in lemon plants. To achieve this, comparative 2-DE and mass spectrometry, in addition to Inductively Coupled Plasma Spectroscopy (ICPS) analyses, were applied to investigate differences in protein accumulation and the concentrations of cationic elements in leaves of healthy and Las-infected lemon plants. Results showed a differential accumulation of 27 proteins, including an increase in accumulation of starch synthase but decrease in the production of photosynthesis-related proteins in Las-infected lemon plants compared to healthy plants. Furthermore, there was a 6% increase (*P* > 0.05) in K concentration in leaves of lemon plants upon Las infection, which support results from previous studies and might represent a common response pattern of citrus plants to Las infection. Interestingly, contrary to reports from prior studies, this study showed a general reduction in the production of defense-related pathogen-response proteins but a 128% increase in Zn concentration in lemon plants in response to Las infection. Taken together, this study sheds light on general and intra-species specific responses associated with the response of citrus plants to Las.

## Introduction

Citrus Huanglongbing (HLB), previously known as citrus greening disease is arguably the most devastating disease threatening citrus production worldwide and all cultivated citrus species are susceptible [1–3]. The disease, which was discovered in Asian countries in the 1870s, has since been identified in other parts of the world including the U.S.A., Brazil, Saudi Arabia and South Africa [3]. ‘*Ca*. Liberibacter asiaticus ’ (Las), a Gram-negative, non-culturable, psyllid-transmissible and phloem-limited α-proteobacterium, is currently the most prevalent causal agent associated with the global presence of HLB [1,3]. Containment of HLB has remained elusive largely due to the paucity of information on the molecular and physiological processes involved in the plant–pathogen interactions associated with the disease.

When microbial pathogens invade a plant, a series of molecular responses are triggered by the plant through defense response pathways in an attempt to protect itself from the pathogenic activities of the microbe. Several reports on plant-microbe interactions suggest that there are highly conserved pathogen-derived molecules, such as flagellin, that generate a generalized or non-host specific molecular response in plants, referred to as nonhost response [4–6]. This response involves an increased production of defense-related proteins such as cysteine proteases, thaumatin-like proteins, chitinases, superoxide dismutase, peroxidases and catalase in the host plants [7]. Additionally, plant-microbe interactions also involves host-to-microbe specific responses in a gene-for-gene manner, whereby a specific pathogen-derived avirulent (*Avr*) protein is recognized by a specific plant resistant (*R*) protein as the case of *Pseudomonas syringae* AvrRPM1 and AvrRpt2 proteins which are respectively recognized by the products of the *Arabidopsis* *RPM1* and *RPS2* resistance genes [8,9]. However, a high-throughput transcriptional analysis study of innate responses of *Arabidopsis* plants showed a strong overlap in the production of nonhost and host-specific proteins in the presence of flagellin, highlighting the power of high-throughput technologies in identifying overall host responses to plant pathogens while noting that understanding plant-microbe interactions is still a complex subject [10].

Intra-species differences in the susceptibility or tolerance of plants to pathogens further complicates the difficulty in understanding plant-microbe interactions as was demonstrated in a transcriptomic study involving ‘Cleopatra’ mandarin ( *Citrus* *reticulata* ) plants and US-897 hybrid ( *Citrus* *reticulata* Blanco x *Poncirus* *trifoliate* ) plants, which are susceptible and tolerant to Las, respectively [11]. Folimonova et al. [12] classified 30 citrus genotypes ranging from sensitive to tolerant according to their response to HLB and two lemon ( *Citrus* *limon* ) varieties “Volkamer” and “Eureka” were classified as moderately tolerant and tolerant, respectively. Additionally, a study by Zhang et al. [13] demonstrated that HLB-affected lemon scions had a higher titer of Las, higher survival rate and pathogen transmission rate than pomelo ( *Citrus* *maxima* ) scions. Although several studies have investigated the molecular mechanisms associated with the response of citrus plants to Las, such studies have largely focused on sweet orange (*Citrus sinensis*) plants [14–19]. Thus, there is limited information in the literature about internal factors associated with the response of other citrus plants particularly those demonstrably tolerant to Las, such as lemon, which could be critical in the development of Las tolerant and resistant citrus varieties to combat HLB.

The importance of sustainable agriculture has become an important topic and there has been a lot of focus on moving away from the classical use of pesticides towards more environmentally-friendly strategies to control plant disease. In particular, it has been suggested that adopting strategic nutritional practices can help reduce plant susceptibility to disease if not completely but at least to a level at which there is a reduced need for other more expensive and less environmentally-friendly practices such as pesticide use [20,21]. Cationic plant nutrients such as Ca, K, Mg, Fe, Cu, Mn, and Zn have been shown to play important roles in plant susceptibility or tolerance to pathogen infection although the effects can sometimes be mixed largely due to the fact that the molecular processes involved in nutrient-disease relationships in plants are not well understood. For example Zn, which is important in the activation of Cu/Zn superoxide dismutase, has been shown to have mixed effects on disease severity by increasing in some cases, while decreasing in other cases, susceptibility to disease [22,23]. Importantly, physiological symptoms of HLB have been suggested to resemble that of Zn-deficiency [24] and the results from a recent study suggests that the availability of Zn will affect the growth of Las in citrus plants [25].

Although high-throughput transcriptional analysis have been successfully used to identify global effects of Las infection on gene expression in citrus plants [11,17,18,26], it is important to note that high-throughput proteomics studies represent the final stage of gene expression and are directly associated with gene expression at a functional level as demonstrated in a recent HLB-related study [15]. A recent proteomics study by our group showed that Las infection induces several defense-related proteins such as lectin-like proteins, miraculin-like proteins, Cu/Zn superoxide dismutase, and chitinases in grapefruit plants and also showed a novel Las-mediated correlated increased-accumulation of granule-bound starch synthase and K in same plants [27]. Since starch synthase requires K for its activation, it is our assumption that this might represent a unique biochemical response of citrus plants to Las infection. To confirm this, a proteomic approach involving 2-DE and mass spectrometry techniques was employed to analyze the protein expression profiles of leaves of healthy or Las-infected lemon plants. Additionally, ICP spectroscopy was applied to investigate the effect of Las-infection on the nutrient status of healthy or Las-infected lemon plants. The goal of this study was to identify a potential consensus pattern in the biochemical responses of citrus plants to Las as well as elucidate interrelationships between protein expression and nutrient concentration levels that could be specifically associated with HLB disease development. Such information would advance our understanding of the molecular and physiological mechanisms involved in host–pathogen interactions.

## Results

### Effect of Las-infection on protein expression

**Figure 1:**
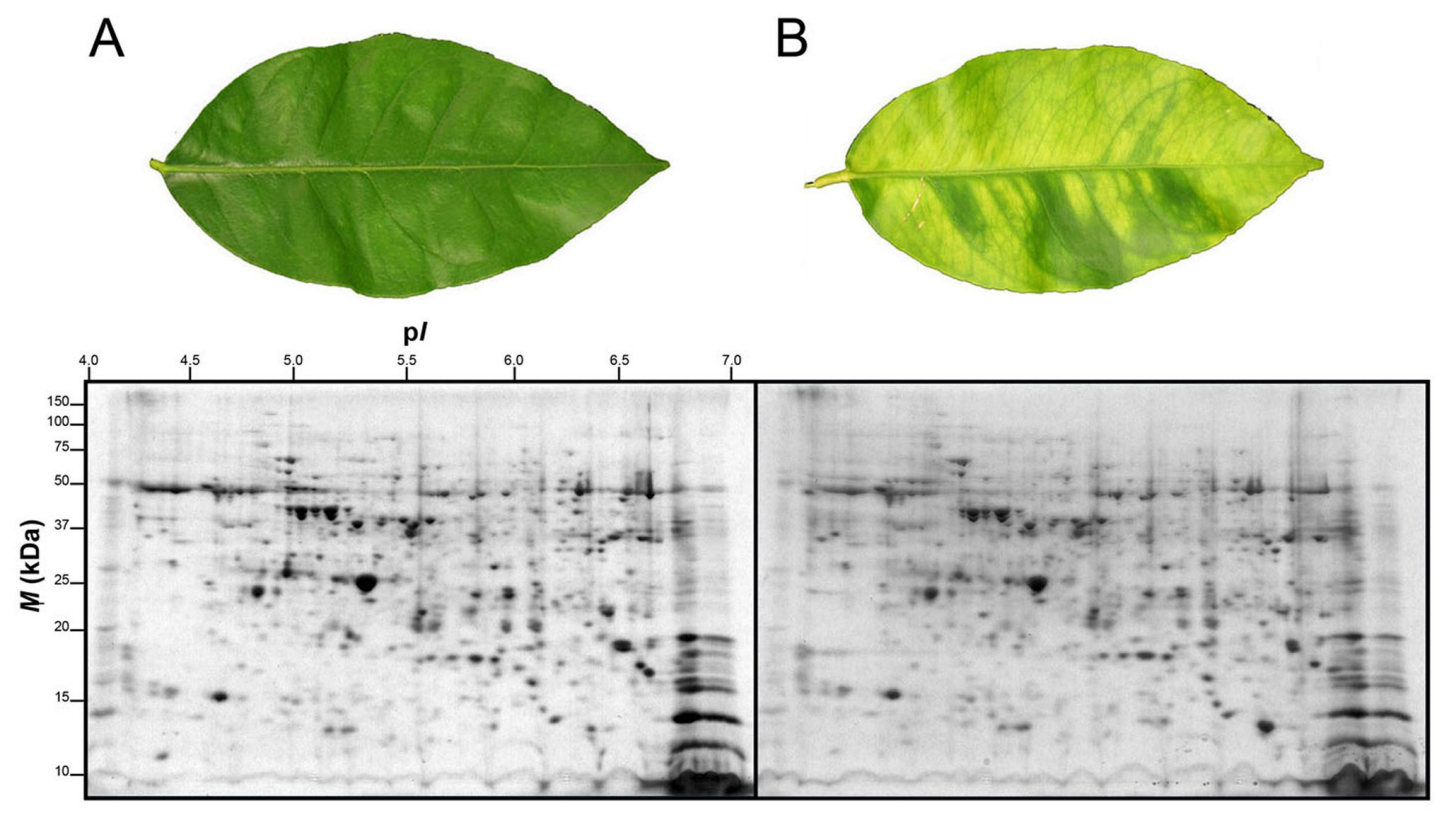
2-DE separation of proteins extracted from leaves of healthy of Las-infected lemon plants. (**A**) Representative leaf and gel containing extracted proteins separated via 2-DE of a healthy lemon plant. (**B**) Representative leaf and gel containing extracted proteins separated via 2-DE of a Las-infected lemon plant. Two-year old healthy plants were either graft-inoculated with side shoots from PCR-confirmed Las-infected bud sticks or uninoculated and leaf samples were analyzed at six months post-inoculation. A sum of 200 µg of total protein was separated according to charge on a pH 4-7 IpG strip and according to mass on 8-16% gradient SDS-polyacrylamide Tris-HCl gels. Protein spots were visualized by staining with Coomassie Brilliant Blue (CBB). *M*_r_, relative molecular mass; pI, isoelectric point.

**Figure 2:**
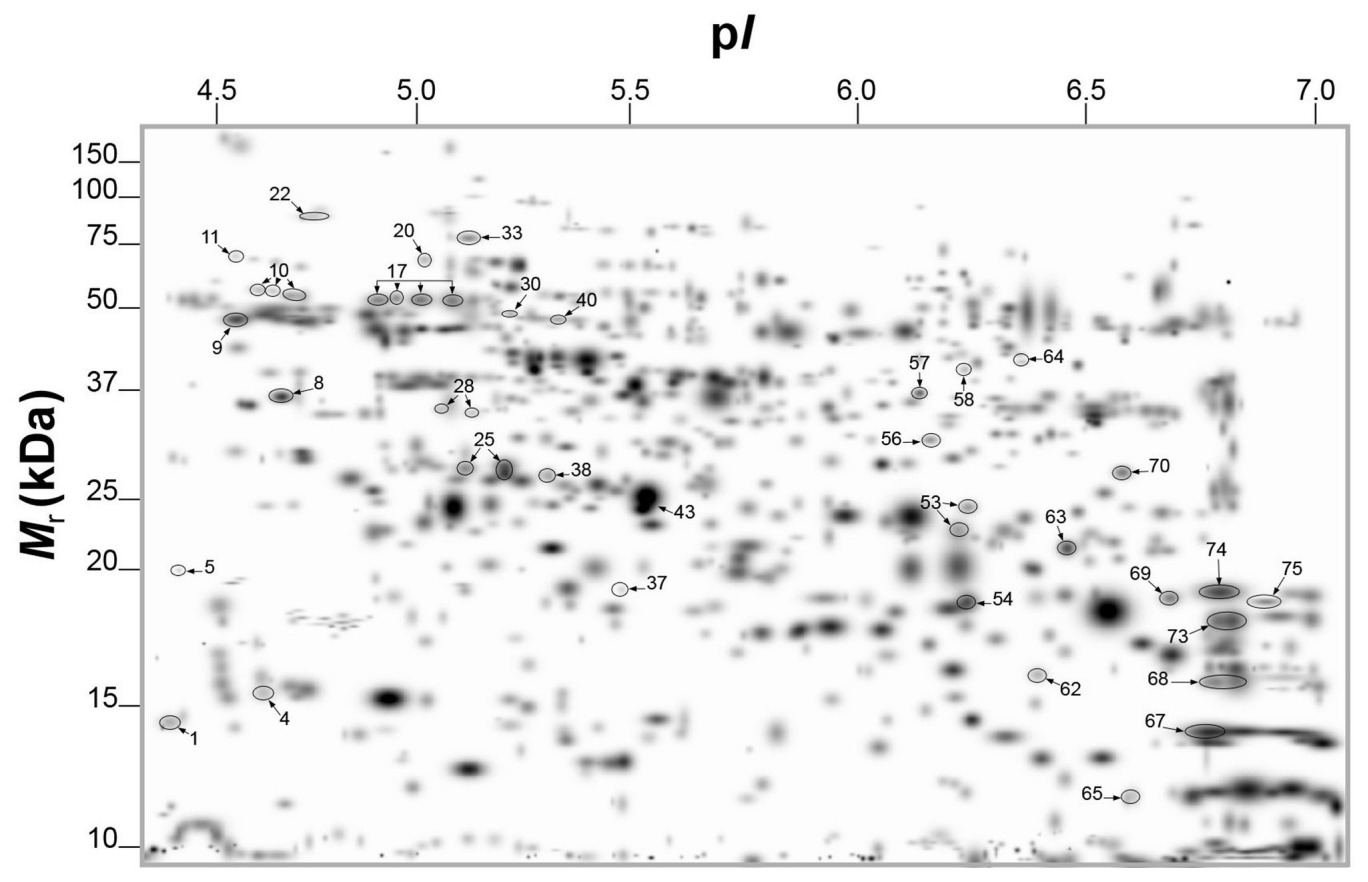
PDQuest-generated master gel image showing the general spot pattern of matched protein spots from the total leaf proteome of healthy or Las-infected lemon plants. Labeled spots were differentially produced in response to Las-infection and described in Table 1. A sum of 200 µg of total protein was separated according to charge on a pH 4-7 IpG strip and according to mass on 8-16% gradient SDS-polyacrylamide Tris-HCl gels. Protein spots were visualized by staining with Coomassie Brilliant Blue (CBB). *M*_r_, relative molecular mass; pI, isoelectric point.

**Figure 3:**
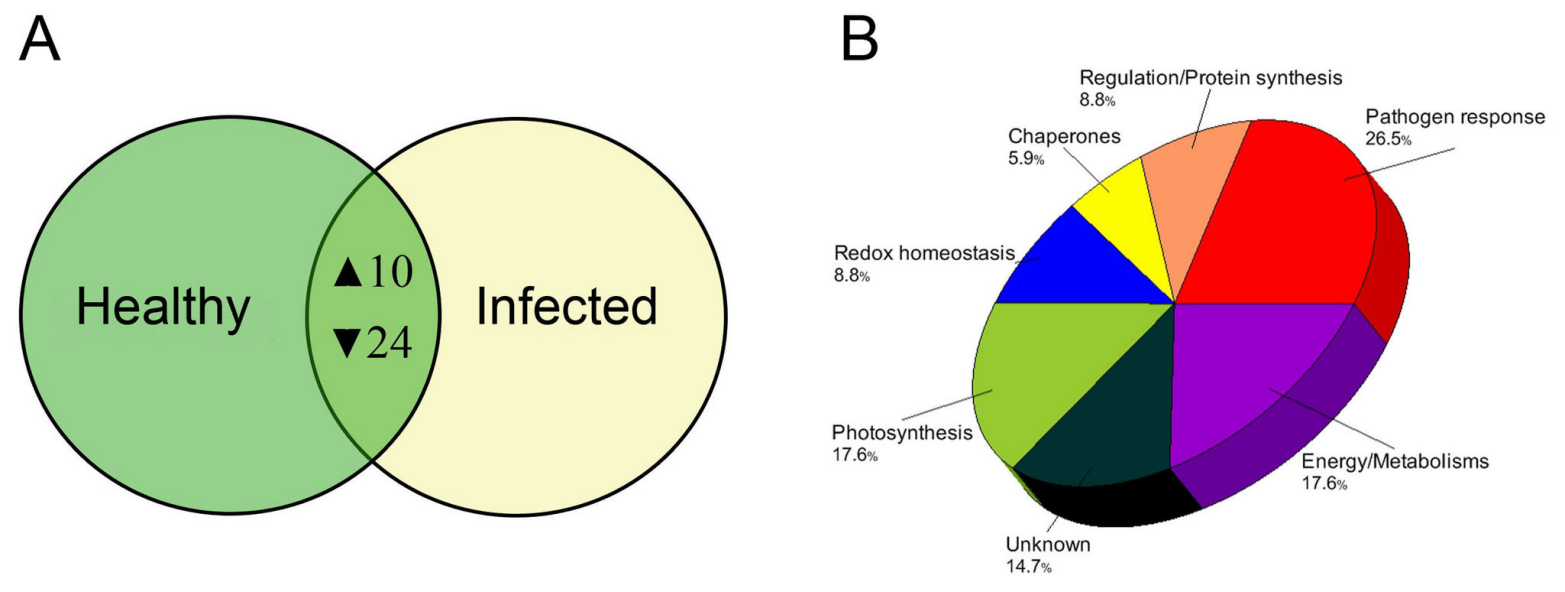
Categorization of differentially produced proteins in lemon plants in response to Las infection. (A) Venn diagram showing the number of protein spots that showed higher-accumulation (▲) or lower-accumulation (▼) in infected lemon plants compared to healthy plants. (B) Functional category distribution of differentially produced protein spots from comparing 2-DE gel images of the total leaf proteome of healthy or Las-infected lemon plants.

**Figure 4:**
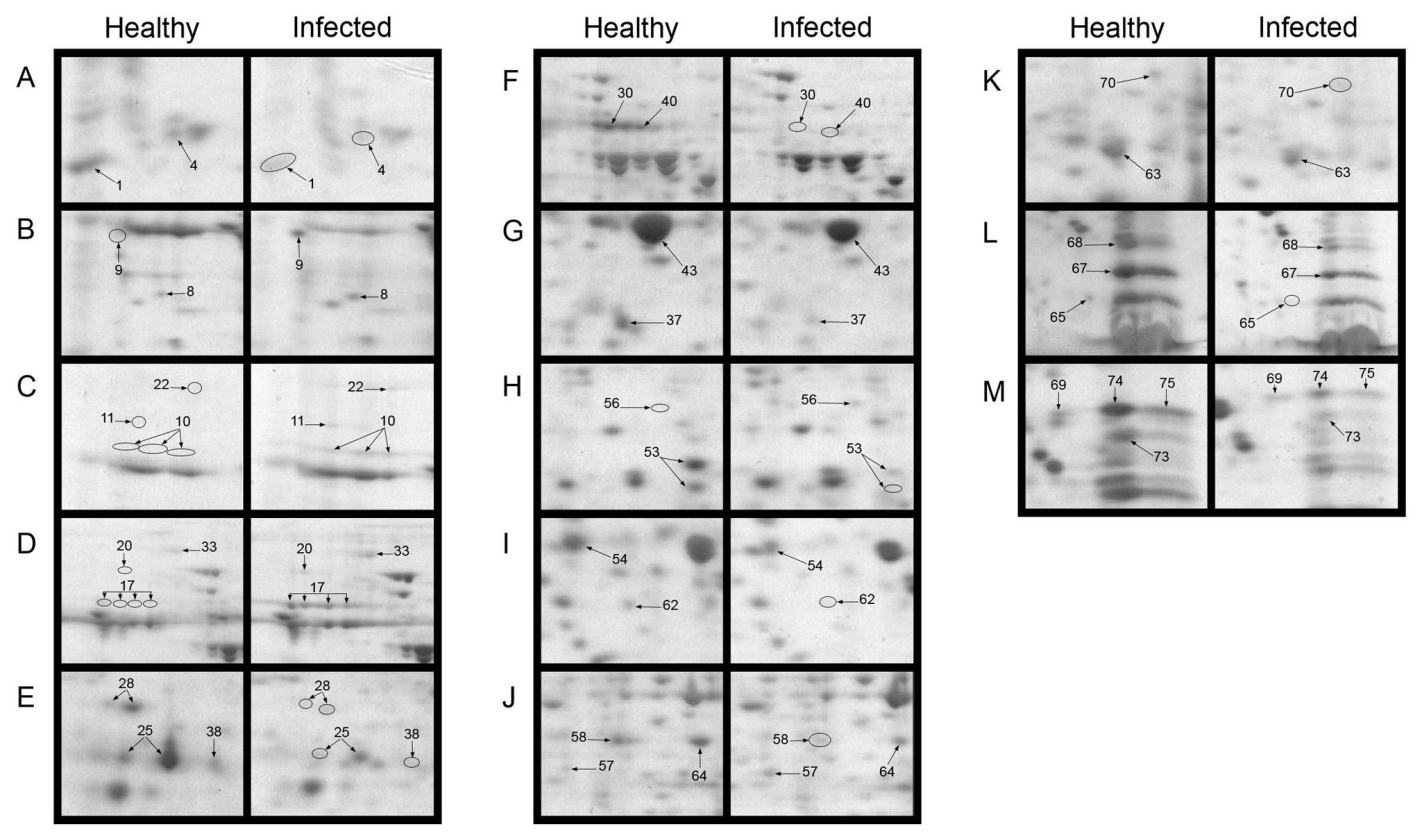
Differentially produced protein spots from 2-DE analysis of total leaf proteins from healthy or Las-infected lemon plants. Panels A-M show magnified views of protein spots in representative 2-DE gels containing separated total proteins from leaves of healthy or Las-infected lemon plants. Labeled spots showed significant changes and correspond to the spots presented in in Figure 2 and Tables 2 and 3. Two-year old healthy plants were either graft-inoculated with side shoots from PCR-confirmed Las-infected bud sticks or uninoculated and leaf samples were analyzed at six months post-inoculation. A sum of 200 µg of total protein was separated according to charge on a pH 4-7 IpG strip and according to mass on 8-16% gradient SDS-polyacrylamide Tris-HCl gels. Protein spots were visualized by staining with Coomassie Brilliant Blue (CBB). *M*_r_, relative molecular mass; pI, isoelectric point.

Leaves of lemon plants infected by Las showed visible HLB-associated symptoms such as blotchy mottling and chlorosis compared to healthy leaves (Fig. 1). However, Las infection had no significant effect on total protein yield in leaves of lemon plants and an average total protein yield of 12.2 mg g^-1^ or 13.9 mg g^-1^ was obtained from leaves of healthy or Las-infected leaves, respectively (Table 1). A high resolution of total protein separation in a pI range between 4 and 7 and molecular mass between 10 and 150 kDa was observed in 2-DE gels from extracted total leaf proteins of healthy or Las-infected lemon plants (Fig. 1). Using PDQuest analysis software we detected over 700 protein spots per gel and over 400 reproducible spots within replicate gels (Table 1). Mass spectrometry analysis via MALDI-TOF- or LC-MS, identified 42 out of 65 protein spots that were differentially produced in response to Las infection. Multiple protein spots matched to the same protein, especially spots around the 65 kDa region and within a pI range of 4.5 and 5.2, which all matched to granule-bound starch synthase. This could be due to a variety of factors including multimerism/protein isoforms, difference in maturation state, degradation and/or post-translational modifications [28,29]. Thus, based on identical protein matches and proximity of spots on gels, the 42 spots identified were summarized into 34 spots (Figure 2). Among these 34 spots, 10 showed higher-accumulation while 24 showed lower-accumulation in Las-infected lemon plants compared to healthy plants (Figure 3A) suggesting an up- or down-regulation of the 10 or 24 protein spots, respectively, due to Las infection. Categorization of proteins according to functional groups showed that majority of the Las-responsive protein spots (26.5%) matched to proteins involved in pathogen response whereas the functions of 14.7% of the identified Las-responsive proteins is unknown (Figure 3B). A magnified view of the profiles of identified spots in representative gels from each treatment group is shown in Figure 4.

**Table 1:** Protein extraction and 2-DE separation result parameters for total leaf proteins from healthy or Las-infected lemon plants.

Parameters	Lemon leaves	
	Healthy	Infected
Protein yield^a^ (mg g^-1^)	12.2 ± 2.0	13.9 ± 2.8
Number of detected spots per gel	763 ± 26	806 ± 23
Number of matched spots in replicate gels	461 ± 38	496 ± 25
Number of matched spots in all gels	188	188

Data represents Means ± SD.

a Protein extraction was repeated three times per sample with three replicate plant samples per treatment.

The expression of stress response-related proteins such as chaperones, redox homeostasis-related proteins and pathogen response-related proteins was significantly affected by Las infection. Interestingly, pathogen response-related proteins such as lectin-related proteins (Table 2, spots 25, 37, and 38), class I chitinase (Table 2, spot 28), and miraculin-like proteins (Table 2, spots 53, 54, 65, 69 and 75) were all down-regulated upon Las infection. Results also showed that catalase (Table 2, spot 30) and a thioredoxin-like protein (Table 2, spot 70), which are involved in redox homeostasis, were down-regulated in Las-infected plants compared to healthy plants. In contrast, chaperones HSP 70 (Table 2, spot 20) and protein disulfide isomerase (spot 33) as well as an isoflavone reductase-related protein (Table 2, spot 56) were up-regulated in lemon plants in response to Las infection.

**Table 2:** Differentially produced proteins in leaves of lemon plants infected with ‘ *Candidatus* Liberibacter asiaticus ’ (Las).

Spot^a^	Δ^b^	Protein function/name^c^		Accession #^c^	Theoretical^d^		S^e^	M^f^	P^g^
					*M*_r_	pI			
	***Pathogenresponse***		
25	-2.18	Lectin-related protein precursor		gi|11596188	29272	5.10	90	8	38
28^h^	-6.99	Acidic class I chitinase		gi|23496445	36735	4.81	52	8	22
37	-4.01	Lectin-related protein precursor		gi|11596188	20359	5.32	69	7	47
38^h^	-2.70	Lectin-related protein precursor		gi|11596188	29272	5.10	61	7	38
53^h^	-5.17	Miraculin-like protein 1		gi|87299375	25585	8.11	61	6	32
54	-3.09	Putative miraculin-like protein 2		gi|119367468	23610	8.18	98	8	45
65	-2.87	Miraculin-like protein 2		gi|87299377	24525	6.88	93	6	32
69	-3.97	Putative miraculin-like protein 2		gi|119367468	23610	8.18	109	7	43
75	-10.71	Putative miraculin-like protein 2		gi|119367468	23610	8.18	110	8	49
	***Chaperones***		
20	On	Heat shock protein 70		gi|6911549	73678	5.10	70	15	29
30	-6.71	Catalase		gi|32526568	57669	6.64	112	15	43
33^h^	1.52	Protein disulfide isomerase		gi|255578860	65701	4.64	60	6	17
	***Redoxhomeostasis***		
56	On	Isoflavone reductase-related protein		gi|3243234	33862	5.92	105	8	40
70^h^	-3.38	Thioredoxin-like protein, chloroplastic		gi|225459760	39336	8.59	56	9	25

a Protein spot numbers are arranged in chronological order and correspond to the numbers given in Figure 1

b Protein fold change in Las-infected leaves compared to healthy leaves; minus sign (-), decrease; On, undetected in healthy leaves.

c Protein function, name and accession number were determined by using http:/www.ncbi.nlm.nih.gov/BLAST/.

d Theoretical molecular mass (*M*_r_) and isoelectric point (pI) were calculated by http:/www.expasy.org/.

e Mascot score.

f Number of matched peptide masses.

g Percent sequence coverage.

h Protein identification confirmed by LC-MS/MS.

^i^ Protein identification confirmed by MALDI-TOF-MS/MS.

A general down-regulation was observed in proteins associated with photosynthesis such as ribulose-1, 5-bisphosphate carboxylase/oxygenase (RuBisCO) (Table 3, spots 1 and 67), oxygen evolving enhancer proteins (Table 3, spots 43, 68, and 74), and a Photosystem (PS) I reaction center subunit II (Table 3, spot 73). On the other hand, there was a significant up-regulation of granule-bound starch synthase (Table 3, spots10 and 17), which is important in starch metabolism. Other metabolism-related proteins that were differentially produced in response to Las infection were aconitate hydratase (Table 3, spot 22), which was up-regulated and a nucleoside diphosphate kinase (Table 3, spot 62), which was down-regulated. Furthermore, proteins potentially involved in gene regulation such as a nucleosome-binding protein (Table 3, spot 9) and a polyadenylate-binding protein (Table 3, spot 11) in addition to glutamine synthase (Table 3, spot 57), which is associated with amino acid synthesis, and an abhydrolase domain-containing protein (Table 3, spot 8) were up-regulated in response to Las infection. However, glutathione transferase (Table 3, spot 63) was down-regulated.

**Table 3:** Differentially produced proteins in leaves of lemon plants infected with Las (Cont’d).

Spot^a^	Δ^b^	Protein function/name^c^		Accession #^c^	Theoretical^d^		S^e^	M^f^	P^g^
					*M*_r_	pI			
	***Photosynthesis***		
1	-2.89	Ribulose-1,5-bisphosphate carboxylase/oxygenase small subunit		gi|24940138	20521	9.16	98	8	42
43	-1.58	Oxygen evolving enhancer protein 1		gi|326467059	29262	5.32	109	13	54
67	-1.64	Ribulose-1,5-bisphosphate carboxylase/oxygenase small subunit		gi|24940138	20521	9.16	116	14	74
68	-1.64	Oxygen-evolving enhancer protein 3-2, chloroplastic		gi|225469185	24927	9.60	109	9	41
73	-2.05	PSI reaction center subunit II		gi|157678730	22606	9.76	104	8	47
74	-3.05	Oxygen-evolving enhancer protein 2, chloroplastic		gi|225446775	26777	8.63	116	7	37
	***Energy/Metabolisms***		
10^h^	On	Granule-bound starch synthase		gi|223029784	67320	8.56
17^h^	8.48	Granule-bound starch synthase		gi|223029784	67320	8.56
22^i^	On	Aconitate hydratase 2		gi|285309969	109307	7.34	41	16	19
62	-1.60	Nucleoside diphosphate kinase, putative		gi|255540363	14819	6.92	89	8	68
	***Regulation/Proteinsynthesis***		
9^i^	On	Nucleosome-binding protein, putative		gi|255553181	36140	4.66
11^i^	On	Polyadenylate-binding protein, putative		gi|255555393	70243	8.09
57	2.63	Glutamine synthase		gi|211906462	39404	5.78	103	11	45
63	-1.52	DHAR class glutathione transferase		gi|283135906	23962	6.18	130	12	62
	***Unknown***		
4	-2.10	Uncharacterized protein		gi|225426158	44041	5.98	79	10	35
5	-4.63	Uncharacterized protein		gi|225426158	44041	5.98	64	11	38
8^h^	1.94	Abhydrolase domain-containing protein		gi|225456828	43339	9.16	47	6	29
40	-1.98	Uncharacterized protein At5g39570-like		gi|356550983	46695	5.00	90	12	37
58	-2.53	Conserved hypothetical protein		gi|255558602	80625	5.65	69	15	30
64	-2.72	Conserved hypothetical protein		gi|255558602	80625	5.65	116	15	29

Legend same as Table 2

### Effect of Las-infection on nutrient status

**Figure 5:**
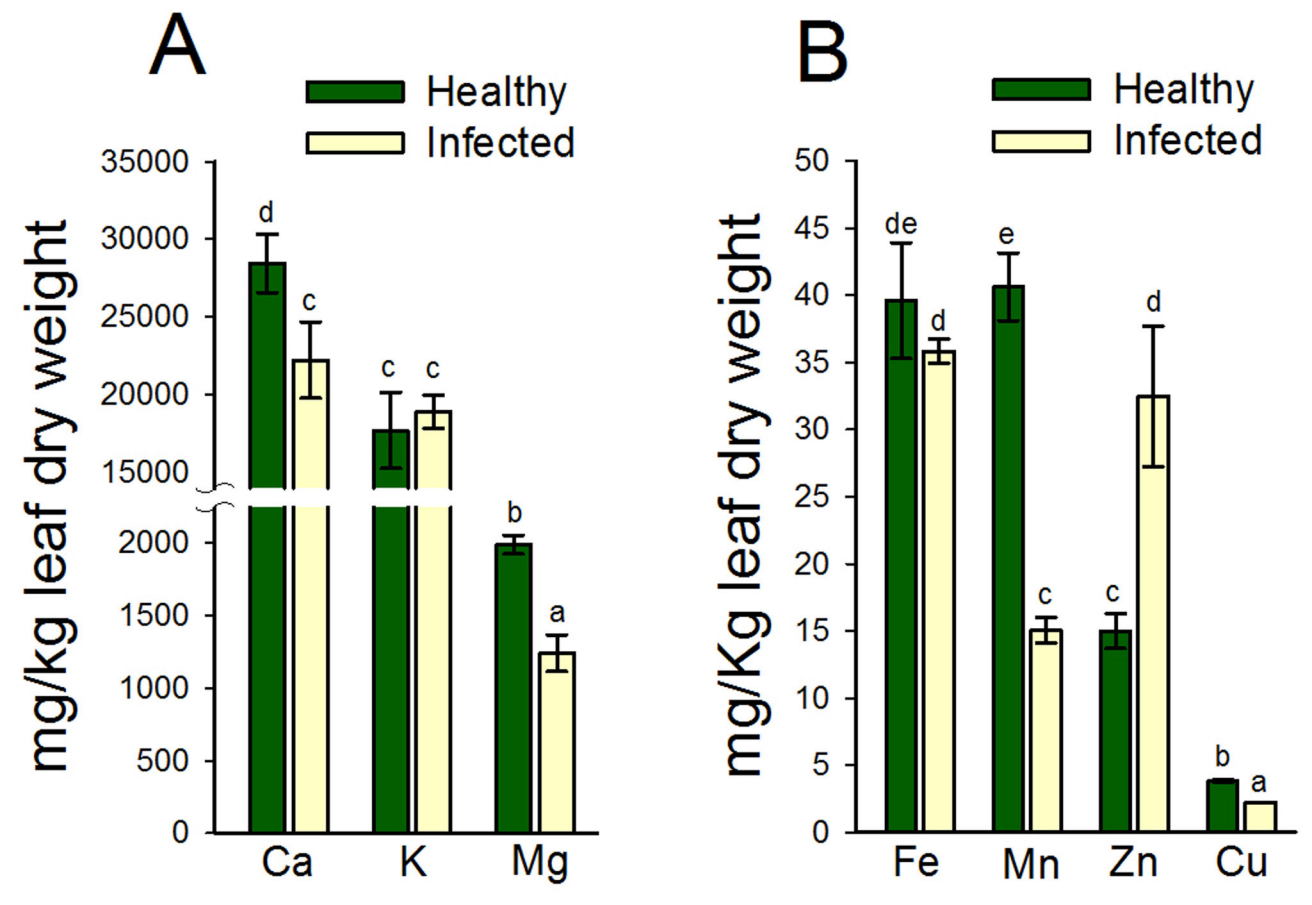
The leaf-nutrient concentrations of healthy or Las-infected lemon plants. (A) Macronutrients: calcium (Ca), potassium (K) and magnesium (Mg); (B) Micronutrients: iron (Fe), manganese (Mn), zinc (Zn), and copper (Cu). Two-year old healthy plants were either graft-inoculated or uninoculated with PCR-confirmed Las-infected bud sticks and leaf samples were analyzed 6 months post-inoculation. Bars within a plant group with the same lower case letter are not significantly different from each other (*P* > 0.05).

Plant cations are actively involved in gene regulation and several metabolically active proteins form co-enzymes with cations. This prompted an investigation on the effects of Las-infection on the nutrient status of lemon plants in tandem with our proteomic analyses. Results showed that there was a 21.8% and 37.6% reduction in Ca and Mg concentrations in leaves of Las-infected lemon plants compared to healthy plants (Figure 5). However, there was a 6% (*P* > 0.05) increase in K concentration in leaves of Las-infected lemon plants compared to healthy plants (Figure 5).

Further analysis of micronutrient concentrations showed that Las-infection resulted in a 9%, 62.9% and 41.3% decrease in the concentrations of Fe, Mn, and Cu in comparison to healthy plants (Figure 5). Interestingly, there was a 128% increase in the Zn concentration of leaves of Las-infected lemon plants compared to healthy plants (Figure 5).

## Discussion

Huanglongbing is a highly destructive disease of citrus and all commercially grown cultivars of citrus species and citrus relatives are susceptible to the disease. Currently, no effective disease control is available largely due to the limited understanding of the molecular and physiological processes associated with the general response of citrus plants to Las infection especially those responsible for intra-species differences in susceptibility [12,13,30,31]. A recent study by our group elucidated novel molecular and nutritional responses of grapefruit plants upon Las infection [27]. Thus, the goal of this study was to identify a potential consensus pattern of biochemical response of citrus plants to HLB by elucidating the molecular and physiological responses of lemon plants to Las infection.

### Las-mediated down-regulation of photosynthesis and pathogen response-related proteins

During periods of biotic and abiotic stress in plants, photosynthesis is typically inhibited and the regulation of gene expression is channeled towards the production of stress-response related factors at the expense of “housekeeping” proteins [32,33]. This is consistent with results from a previous study by Albrecht and Bowman on sweet orange plants [26] as well as results from our recent study on grapefruit plants [27], which showed a Las-mediated down-regulation of photosynthesis-related gene transcripts and photosynthesis-related proteins, respectively, as part of the molecular mechanisms associated with citrus response to HLB. These same studies and two others on citrus plants also showed a Las-mediated up-regulation of defense-related transcripts or proteins, such as catalase, chitinase, lectin-related proteins and miraculin-like proteins [15,17,26,27], suggesting that a Las-mediated down-regulation of photosynthesis but up-regulation of defense-response processes could represent a consensus pattern of response of citrus to HLB.

Thus, it was not surprising that all photosynthesis-related proteins identified in our current study were down-regulated in lemon plants in response to Las infection (Table 3). However, except for HSP70, protein disulfide isomerase and an isoflavone reductase-related protein, which were up-regulated, all other stress/pathogen-response related proteins identified in this study such as catalase, chitinase, lectin-related proteins and miraculin-like proteins were markedly down-regulated in lemon plants upon Las infection (Table 2). This might suggest a pathogenicity scheme for Las in lemon plants since prior studies have shown that microbial pathogens can suppress host defense response processes to facilitate invasion. Hauck et al. [34] demonstrated that *Pseudomonas syringae* type III secretion system-related AvrPto protein suppresses or down-regulates the expression of a set of *Arabidopsis* genes that encode putative cell wall and defense proteins in a salicylic acid-independent manner. Additionally, Bouarab et al. [35] showed that the fungal pathogen *Septoria* *lycopersici* produces tomatinase, which degrades saponin in *Nicotiana* *benthamiana* and suppresses the production of defense response proteins.

Catalase, chitinase, lectin-related proteins and miraculin-like proteins form part of the nonhost or innate response of plants to pathogens and it is unclear why all of these important defense-related proteins are down-regulated in lemon plants in response to Las infection. However, we suggest that since these proteins are not specific for Las, reducing their expression levels could be part of an energy conservation mechanism for an efficient utilization of resources by lemon plants upon Las-induced stress. Additionally, lectin-like proteins play a role in vascular tissue differentiation [36] and are involved in the plugging of phloem sieve plates in response to wounding and defense against pathogens and insects [37]. Thus, while lectin-mediated phloem-blockage might help limit bacterial spread, it could also limit the flow of photosynthates to other parts of the plant, potentially making those malnourished parts more vulnerable to disease. It appears that response to Las in lemon plants involves a scenario whereby the sieve-tubes are not clogged, which facilitates the flow of photosynthates. This is supported by a recent study by Fan et al. [16] that demonstrated that phloem transport was less inhibited in rough lemon leaves compared to sweet orange leaves. However, this could also enhance systemic spread and higher titers of Las in lemon tissues as was shown by Zhang et al. [13].

### Las-mediated up-regulation of starch synthase and aconitate hydratase in lemon plants

This study identified 10 protein spots that were up-regulated in lemon plants in response to Las-infection, which included multiple isoforms of granule-bound starch synthase around the 65 kDa region and within a pI range of 4.5 and 5.2. The increased-accumulation of starch in plant tissues during HLB disease development has been previously demonstrated [38,39], but the molecular mechanisms involved are yet to be resolved.

Starch biosynthesis is controlled by four major enzymes namely: ADPase, starch synthase, granule-bound starch synthase, and starch debranching enzyme. ADPase catalyzes the rate limiting interconversion of glucose-1-phosphate to ADP-glucose. ADP-glucose is then polymerized into amylopectin by multiple isoforms of starch synthase or to amylose by granule-bound starch synthase [40]. Starch debranching enzyme together with starch branching enzyme regulates the extensive branching of amylopectin. Transcriptomics studies by Albrecht and Bowman [26] and Kim et al. [17] showed that the most up-regulated starch anabolism-related gene transcript in HLB-affected sweet orange (*Citrus sinensis*) plants was ADP-glucose pyrophosphorylase (ADPase). However, at the protein level, the fact that our recent study on grapefruit plants [27] and this study on lemon plants only showed a significant increase in the accumulation of granule-bound starch synthase compared to other starch biosynthesis-associated enzymes, suggests that the enzyme might play a key post-transcriptional role in HLB-mediated increase in starch accumulation in citrus plants. It is also important to mention that the occlusion of phloem tubes by aggregation of Las bacteria can result in the accumulation of starch. Additionally, the accumulation of starch could result in an inhibition of photosynthesis via a negative feedback mechanism.

Besides granule-bound starch synthase, another metabolism-related protein that was up-regulated in response to Las-infection was aconitate hydratase 2 (Table 3). Aconitate hydratase 2 is a mitochondrial enzyme with a citrate hydro-lyase catalytic activity, which is involved in several metabolic processes especially the reversible isomerization of citrate and isocitrate as part of the tricarboxylic acid (TCA) cycle. An up-regulation of aconitate hydratase was shown in rice (*Oryza sativa*) plants under Cd stress [41] and in *Arabidopsis thaliana* seedlings under phosphate deficiency [42]. Durand et al. [43] and Kieffer et al. [44] showed a patterned response to stress in poplar plants, whereby respiration and glucose catabolism-related proteins are up-regulated while proteins involved in photosynthesis are down-regulated. Consistent to their results, we observed a general down-regulation of photosynthesis-related proteins in addition to an up-regulation of aconitate hydratase in response to Las-infection (Table 3). Although the mechanisms involved in this patterned-response are not well understood, López-Millán et al. [45] suggested that a stress-mediated slowing down of photosynthetic carbon fixation would limit carbon availability, which might cause plants to induce processes involved in carbohydrate catabolism resulting in a remobilization of carbon/energy storage compounds.

### Las-mediated up-regulation of regulatory and amino acid synthesis-related proteins in lemon plants

An up-regulation of a nucleosome-binding protein and a polyadenylate-binding protein was observed in Las infected plants compared to healthy plants (Table 3). Nucleosome-binding proteins and polyadenylate-binding proteins are regulatory proteins, which are directly involved in DNA replication, transcription, and transcriptional/post-transcriptional modification of gene expression [46,47]. Although reports on pathogen/biotic stress-mediated up-regulation of these proteins in plants is limited, under stress conditions, High Mobility Group B (HMGB) proteins, which are nuclear proteins that can bind to nucleosomes in a non-sequence specific manner, are known to be secreted into the extracellular milieu and function as pro-apoptotic proteins [47]. In addition, Minard et al. [48] showed that Asf1, a nucleosome-interacting protein in yeast, promotes the expression of DNA damage response genes during the S phase. Kozubowski et al. [49] showed that Pub1, a poly (A)-binding protein, is a subunit of the catalytic complex of calcineurin, which is a calcium-calmodulin-activated phosphatase important in cellular responses to stress. Thus, it should be promising to further investigate and characterize the putative nucleosome-binding protein and putative polyadenylate-binding protein identified in this study, especially when they represent the first observation of a potential involvement in the response of citrus plants to Las-infection.

Glutamine synthase, which catalyzes the production of glutamine from glutamate and ammonia and is actively involved in nitrogen metabolism, was also up-regulated in response to Las-infection. Purcino et al. [50] showed a significant up-regulation of glutamine synthase in sweet orange (*Citrus sinensis* cv. Pera) plants infected with the bacterium, *Xyllela* *fastidiosa* , and further showed that a disturbance in the nitrogen metabolism of the host plant could play a role in disease development.

### Las-mediated modulation of nutrient homeostasis in lemon plants

Disease symptoms typically reflect the altered nutritional status of plants and nutrient-disease interactions in plant systems are well documented [20]. A malfunctioning or blocked vascular system such as that implicated in HLB-disease development [17,26] can induce a systemic or localized nutrient deficiency or sufficiency, respectively. Nonetheless, diseased plants have generally been shown to have reduced nutrient concentrations compared to healthy plants [20], which is supported by our observation of reductions in the concentrations of Ca. Mg, Fe, Mn and Cu in Las-infected lemon plants compared to healthy plants (Figure 5).

Plant nutrients are actively involved in gene regulation and several metabolically active proteins depend on the availability for specific nutrients for activation. Starch synthase depends on K for its activation and in this study, we observed a Las-mediated 6% increase (*P* > 0.05) in K concentration as well as an increase in the accumulation of starch synthase in lemon plants. This is consistent with results from our earlier study that showed a coordinated increase in K concentration and starch synthase accumulation in leaves of Las-infected grapefruit plants compared to healthy plants [27]. Pathogen-mediated disruption of membrane permeability could lead to electrolyte leakage [20], which could represent a virulence mechanism by Las to induce host K accumulation to sustain increased starch production, thus providing a steady source of carbon and other nutrition for this phloem-limited bacterium while the host cells are deprived of these nutrients.

Furthermore, symptoms of HLB are very similar to those of Zn-deficiency and citrus plants have generally shown reduced concentrations of Zn in tissues due to Las infection [3,24,51]. It was therefore surprising to see a 128% increase in Zn concentrations in lemon leaves in response to Las infection (Figure 5), especially when further investigations of the nutritional content of other citrus and citrus-related plants showed a reduction in leaf-Zn concentrations (unpublished data). Additionally, a recent study involving ‘Navel’ sweet orange scions on Cleopatra mandarin rootstocks, showed a slight increase (*P* > 0.05) in Zn concentration in leaves due to Las infection [19]. Together, these observations suggest that the effect of Las on the nutritional status of citrus plants remains unresolved and the intra-species differences in nutrient homeostatic response to Las infection could play a role in differences in susceptibility to Las [12,13].

The accumulation of metals, such as Zn, has been suggested to be part of an “elemental defense” mechanism in plants [52]. Fones et al. [53] showed a close correlation between the accumulation of Zn, nickel (Ni), and cadmium (Cd) in leaves of *Thlaspi* *caerulescens* plants and the resistance of such plants to bacterial leaf spot caused by *Pseudomonas syringae* pv. maculicola. Coleman et al. [54] investigated the relative toxicities of eight metals, including Zn, which are commonly accumulated or hyperaccumulated by plants and indicated that the elemental defenses provided by metal accumulation in plants can be effective at concentrations far lower than previously hypothesized. It seems that the Las-mediated increase in Zn concentration could be complementary to the observed and earlier discussed Las-mediated down-regulation of defense-related proteins in lemon plants. Thus, when we combine our observation of a lack of a Las-mediated up-regulation in defense-related proteins with that of our observation of a Las-mediated 128% increase in Zn concentration in lemon leaves, it is tempting to propose that the active defensive response of lemon plants to Las infection might be more focused at the nutritional level rather than at the proteomic level.

## Conclusions

This study showed that Las infection resulted in a down-regulation of photosynthesis-related proteins but an up-regulation of granule-bound starch synthase accompanied by a 6% increase (*P* > 0.05) in K concentration of lemon plants. All of which is consistent with reports from prior studies and might be part of a consensus pattern of biochemical response of citrus plants to Las infection. Additionally, the study identified potential intra-species specific responses of citrus to Las, particularly an observation that Las-infection facilitates a down-regulation of defense-related proteins accompanied by a 128% increase in Zn concentration in lemon plants. An interesting find that might provide information on the potential biochemical mechanisms associated with the Las-induced responses in lemon plants, which have been previously shown to display a significant level of tolerance to Las. Thus, the information provided in this study has shed more light on the molecular and physiological mechanisms involved in host response to HLB, which could be applicable towards (i) development of citrus plants with reduced susceptibility to Las, (ii) development of more efficient nutritional management programs to control the disease, (iii) a broader understanding of plant-microbe interactions.

## Materials and Methods

### Growth conditions and treatments

Plant growth was performed under controlled conditions in an insect-proof greenhouse at the U.S. Horticulture Research laboratory, U.S. Department of Agriculture, Fort Pierce, Florida. Two-year old Lemon plants ( *C* *. limon* cv. ‘Todo del Ano’ grafted onto *C* *. paradisi* cv. ‘Duncan’ rootstock) from the same progeny were either uninoculated or inoculated by side-grafting with 3-4 cm long PCR-confirmed HLB-affected lemon bud sticks [13]. The absence or presence of Las in plants, pre- or post-inoculation, respectively, was confirmed by quantitative real-time PCR as previously described [55]. Inoculated plants with confirmed Las presence were henceforth described as Las-infected.

Plants were arranged randomly on the greenhouse bench and kept under natural light conditions at a temperature of 23–30 ^°^C. Plants were irrigated as needed and fertilized every three weeks using a water-soluble fertilizer mix, 20N-10P-20K (Peters Professional, The Scotts Company, Marysville, OH). Micronutrients (Micro Key Palm and Ornamental Formulation, Brandt Consolidated, Springfield, IL) and additional iron (Sequestrene 138 Fe, Becker Underwood, Ames, IA) were applied. Plants were pruned immediately after graft-inoculation to promote new leaf growth and HLB disease development.

Six months post-inoculation, 10-15 fully expanded leaves were collected from three individual plants each from the healthy or infected group of plants. At this stage the infected plants were symptomatic (blotchy mottle and yellow shoots) and PCR-positive for Las. Harvested leaves were immediately frozen in liquid nitrogen and stored at -80 ^°^C until further analysis.

### Protein extraction and quantification

The method used for total leaf protein analysis was modified after Nwugo and Huerta [41]. Leaves from individual plants were pooled and ground to a fine powder in liquid nitrogen using a freezer mill (6850 Freezer/Mill, Wolf Laboratories Ltd., UK). Approximately 0.4 g of leaf powder was transferred to sterile 5 mL polyallomer centrifuge tubes (Beckman Instruments Inc., USA) and suspended in 4.5 mL of chilled solution A [90% (v/v) acetone, 9.9993% (v/v) trichloroacetic acid (TCA), 0.0007% (v/v) Beta-mercaptoethanol]. The mixture was incubated overnight at -80 ^°^C followed by centrifugation at 4 ^°^C for 20 min at 36,000 *g* (Optima L-70K Ultracentrifuge, Beckman Coulter Inc., USA). The supernatant was decanted, and the pellet was washed at least three times until the supernatant was clear (not greenish) by resuspension in 4.5 mL of chilled solution B [98.53% (v/v) acetone, 1 mM polymethylsulphonylfluoride (PMSF), 2 mM EDTA, 0.0007% (v/v) Beta-mercaptoethanol], incubation for 1 h at -80 ^°^C followed by centrifugation at 4^°^C for 20 min at 36,000 *g*. The whitish pellet or crude protein extract was then transferred into sterile eppendorf tubes and vacuum-dried (Vacufuge™, Eppendorf, Germany). The dry pellet, which could be stored indefinitely at -80 ^°^C, was suspended in 0.5mL of rehydration/isoelectric focusing (IEF) buffer [8 M Urea, 50 mM DTT, 4% (w/v) CHAPS, 0.2% (v/v) 3/10 ampholytes, 0.002% (w/v) bromophenol blue] and incubated at room temperature (RT) for 30 min to solubilize proteins. Insoluble material was removed by centrifugation at RT at 14,000 *g* for 15 min and 5µL of the supernatant was prepared using the Compat-Able™ Protein Assay Preparation Reagent Set (Pierce, Rockford, IL, USA) for total protein quantification via bicinchoninic acid (BCA) assay (Pierce, Rockford, IL, USA). Total protein extraction and quantification process was repeated three times generating three analytical replicates per plant.

### 2-DE separation and image analysis

For first dimension electrophoresis or IEF, 11-cm long pH 4-7 ReadyStrip IPG strips (Bio-Rad, Hercules, CA, USA) were passively rehydrated overnight at RT with 0.2 mL of IEF buffer containing 1mg/mL of total solubilized proteins. Rehydrated strips were placed in a PROTEAN IEF cell (Bio-Rad) and IEF was performed at a current limit of 50 µA/per IpG strip at 10 ^°^C, in the following steps: active rehydration at 250 V for 9 h; 250 V (linear) for 15 min; 8 kV (linear) for 3 h; and 10 kV (rapid) until a total 60 kVh for a combined total of approximately 70 kVh. Each focused IPG strip was equilibrated by soaking, with mild stirring, in 4 ml of equilibration base buffer 1 (EBB1) [8M urea, 2% (w/v) sodium dodecyl sulphate (SDS), 50 mM Tris-HCl (pH 8.8), 20% (v/v) glycerol, 1% (w/v) DTT] for 10 min, followed by soaking in 4 ml of EBB2 [same content as EBB1 except DTT was replaced with 2.5% (w/v) iodoacetamide (IAA)]. Second dimension electrophoresis was performed in 8-16% gradient SDS-polyacrylamide Tris-HCl gels (Criterion precast gels, Bio-Rad) in a twelve-gel cell system (Criterion Dodeca Cell, Bio-Rad). Protein spots were visualized by staining with Biosafe Coomassie. Stained gels were scanned (ScanMaker 9800XL, Microtek, USA) under identical conditions and stored in 0.02% NaN_3_ at 4 °C.

Gel images were analyzed using the PDQuest software package (version 8.0, Bio-Rad, USA). A total of 18 gels were analyzed representing three analytical replicates per plant and three replicate plants per treatment. The gels were assigned to two groups namely: healthy or infected. Gel spots were detected and matched so that a given spot had the same number across all gels. A master gel image containing matched spots across all gels was auto-generated. Extensive analysis using the “Landmark” tool was used to resolve missed matches and spot volumes were normalized according to the total gel image density as suggested by the PDQuest software package. An average spot volume was determined for each spot per group and pair-wise quantitative as well as statistical analysis sets were generated by comparing the average volume of a given spot between both treatments. Only spots that had ≥10-fold increase over background and present in at least six of the nine gels per treatment as well as showed ˃1.5 fold change (*P* < 0.05) compared to the other treatment group were considered to be differentially produced and further analyzed.

### Trypsin digestion and mass spectrometry

Protein spots were manually excised (OneTouch Plus Spotpicker, The Gel company, USA), reduced with DTT, alkylated with IAA, and digested with mass spectrometry grade trypsin in the presence of ProteaseMAX^™^ Surfactant according to the manufacturer’s protocol (Promega, USA). Acetonitrile extraction was used to enhance peptide recovery. Tryptic-digests were generally analyzed by MALDI-TOF- or LC-MS/MS.

For MALDI-TOF-MS or MS/MS analysis (QSTAR XL Hybrid Quadrupole TOF LC/MS/MS System, Applied Biosystems, USA), the target plate was spotted with 2 µL of a 1:1 (v/v) mixture of tryptic-digest and matrix solution [10 mg/mL α-cyano-4-hydroxycinnamic acid (CHCA) in 50% ACN/ 0.1% TFA]. Mass spectra were acquired in positive TOF MS mode over the mass range of 800–4000 Da using 300 one-second cycles with MCA on. A mixture of Des-Arg1-Bradykinin (904.47), Angiotensin I (1296.68), Neurotensin (1672.92) and ACTH (2093.09, 2465.20, 3657.92) monoisotopic [M+H]^+^ mass standards (Anaspec, USA) were used for external calibration. Monoisotopic peaks with S/N >5 were selected as the peptide mass fingerprint (PMF) per spot. Parent ion spectra (MS/MS) was acquired over a mass range of 50–4000 Da using 300 one-second cycles with MCA on.

For LC-MS/MS analysis (Ultimate 3000 RLSCnano System linked to Velos LTQ Orbitrap, Thermo, Fisher), peptides were solubilized in 0.1% TFA and loaded on to a self-made fused silica trap-column of 100 µm X 2 cm packed with Magic C18 AQ (5µm bead size, 200Å pore size Michrom Bioresources, Inc.) and washed with 0.2% formic acid at a flow-rate of 10 µL/min for 5 min. The retained peptides were separated on a fused silica column of 75 µm X 50 cm self-packed with Magic C18 AQ (3µm bead size, 200Å pore size, Michrom Bioresources, Inc.) using a linear gradient from 4 to 45% B (A: 0.1% formic acid, B: 0.08% formic acid, 80% ACN) in 30 min at a flow-rate of 300 nL/min. For each cycle, one full MS was scanned in the Orbitrap with resolution of 60000 from 300–2000 m/z followed by CID fragmentation of 20 most intense peaks. Data dependent acquisition was set for repeat count of 2 and exclusion of 60 sec.

### Protein identification via database queries

Prior to database queries, the Peak Erazor software (v 2.01: Lighthouse data, Odense, Denmark) was used to process peptide mass fingerprints (PMFs) generated from MALDI-TOF-MS analysis as previously described [41]. The MASCOT search engine (Matrix Science, London, UK) was used to find matches of the PMF and MS/MS fragmentation spectra against a custom database containing entries for citrus (*Citrus sinensis* and *Citrus Clementina*) available at http://www.citrusgenomedb.org/ and entries for grape (*Vitis vinifera*) available in the NCBI nonreduntant database. The PAC nos. for citrus or Accession nos. for grape entries that matched to our protein/peptide queries at the moment of Mascot search was recorded. Fixed and variable modifications (Cys carbamidomethylation and Met oxidation, respectively) and one missed cleavage were considered. PMF database search was conducted using a maximum mass tolerance of ±100 ppm, while MS/MS ions search were conducted with a mass tolerance of ± 0.6 Da on the parent and 0.3-0.8 Da on fragments; in all cases the peptide charge was +1. Decoy search was done automatically by Mascot on randomized database of equal composition and size. For PMF analysis, the peptide mixtures that produced the highest statistically significant (*P* < 0.05) match scores and accounted for the majority of the peaks present in the mass spectra, were assumed to be positively identified proteins.

LC-MS/MS spectra were also searched via MASCOT against a custom citrus database using the following parameters: precursor mass tolerance 10 ppm, fragment mass tolerance: 0.6 Dalton, fixed modification of carbamidomethylaion on cysteine and variable modification of methionine oxidation. The peptide identification results were filtered using a False-Detection-Rate (FDR) of 1% and only the top match was reported. To gain functional information on identified proteins from MALDI-TOF and LC-MS/MS analysis, homology searches using BLAST_P_ (http:www.ncbi.nlm.nih.gov/BLAST) was employed.

### Nutrient Status analysis

The same samples used for proteomic analysis were assayed for major cationic elements, Ca, K, Mg, Fe, Cu, Mn, and Zn via Inductively-Coupled Plasma Optical Emission Spectroscopy (ICP-OES) as previously described [56]. Briefly, leaf tissues were oven-dried and 0.5g was ashed at 510 ^°^C for 9hrs, allowed to cool, and digested in 10 mL of 1N HNO_3_ for 1 h. The filtered supernatant was brought to volume (25 mL) and the intensities of atomic emissions at 396.847nm for Ca, 766.491nm for K, 279.553nm for Mg, 238.204nm for Fe, 327.395nm for Cu, 257.610nm for Mn, and 213.857nm for Zn was measured on an ICP-OES System (Varian, Vista Pro CCD Simultaneous ICP-OES attached to Varian SPS 5 Sampler Preparation System, Agilent, USA). Samples were further diluted 1:100 in 1N HNO_3_ prior to analysis of macronutrients Ca, K, and Mg while the analysis of micronutrients Fe, Cu, Mn and Zn did not require further dilutions. All containers used for ICP Spectroscopy analysis were acid-washed by soaking overnight in 1N HNO_3_ before use.

### Statistical analysis

The nutrient concentration data were subjected to analysis of variance (ANOVA) using SigmaPlot software Version 11 (Systat Software, Inc., Point Richmond, California, USA) and means were separated using the Fischer’s Least Significant Difference (FLSD) test at ˃ 95% confidence interval (*P* < 0.05). Pair-wise comparisons to determine significant differences in spot volumes between treatments were performed on standardized log_10_ values of protein spot volumes using the Student’s *t*-test analysis at ˃ 95% confidence interval (*P* < 0.05) as provided by the PDQuest software.
